# Identification and Characterization of 5′ Untranslated Regions (5′UTRs) in *Zymomonas mobilis* as Regulatory Biological Parts

**DOI:** 10.3389/fmicb.2017.02432

**Published:** 2017-12-08

**Authors:** Seung Hee Cho, Katie Haning, Wei Shen, Cameron Blome, Runxia Li, Shihui Yang, Lydia M. Contreras

**Affiliations:** ^1^Institute for Cellular and Molecular Biology, University of Texas at Austin, Austin, TX, United States; ^2^Department of Chemical Engineering, Cockrell School of Engineering, University of Texas at Austin, Austin, TX, United States; ^3^Hubei Key Laboratory of Industrial Biotechnology, Hubei Collaborative Innovation Center for Green Transformation of Bio-resources, Environmental Microbial Technology Center of Hubei Province, College of Life Sciences, Hubei University, Wuhan, China

**Keywords:** regulatory RNA, 5′ UTR, *Zymomonas mobilis*, 5′ rapid amplification of cDNA ends (RACE), stress response

## Abstract

Regulatory RNA regions within a transcript, particularly in the 5′ untranslated region (5′UTR), have been shown in a variety of organisms to control the expression levels of these mRNAs in response to various metabolites or environmental conditions. Considering the unique tolerance of *Zymomonas mobilis* to ethanol and the growing interest in engineering microbial strains with enhanced tolerance to industrial inhibitors, we searched natural *cis*-regulatory regions in this microorganism using transcriptomic data and bioinformatics analysis. Potential regulatory 5′UTRs were identified and filtered based on length, gene function, relative gene counts, and conservation in other organisms. An *in vivo* fluorescence-based screening system was developed to confirm the responsiveness of 36 5′UTR candidates to ethanol, acetate, and xylose stresses. UTR_ZMO0347 (5′UTR of gene ZMO0347 encoding the RNA binding protein Hfq) was found to down-regulate downstream gene expression under ethanol stress. Genomic deletion of UTR_ZMO0347 led to a general decrease of *hfq* expression at the transcript level and increased sensitivity for observed changes in Hfq expression at the protein level. The role of UTR_ZMO0347 and other 5′UTRs gives us insight into the regulatory network of *Z. mobilis* in response to stress and unlocks new strategies for engineering robust industrial strains as well as for harvesting novel responsive regulatory biological parts for controllable gene expression platforms in this organism.

## Introduction

Biomass pretreatment and hydrolysis releases sugar monomers from cellulose and hemicellulose. Several growth inhibitors are released from this process including xylose and acetate (Doran-Peterson et al., 2008; Mohagheghi et al., 2014). *Zymomonas mobilis* is a promising ethanologenic bacterium due to its efficient ethanol production and high ethanol tolerance (16% v/v). Several recent reviews have outlined the progress that has been made in understanding ethanol-related pathways in this organism (Rogers et al., 2007; He et al., 2014; Yang et al., 2016a). Originally, wild-type *Z. mobilis* could only utilize glucose, sucrose, and fructose as carbon sources for ethanol production, but metabolic engineering approaches have enabled xylose and arabinose metabolisms (Zhang et al., 1995, 2007; Morris and Mattick, 2014).

Transcriptomic and proteomic analyses of *Z. mobilis* in ethanol-supplemented conditions revealed that genes associated with DNA repair, membrane biogenesis, carbohydrate metabolism, transport, and transcriptional regulation are differentially expressed in ethanol stress, showing the complexity of this phenotype (He et al., 2012a; Yang et al., 2013). Additional omics studies have shown both xylose and acetate as important inhibitory factors of *Z. mobilis* growth and ethanol production (Yang et al., 2014a). Upon co-utilization of xylose and glucose, especially with inhibitors such as acetate or furfural, the gene expression of redox mechanisms and carbon and energy metabolisms are dramatically changed (He et al., 2012b; Yang et al., 2014a). While it is well-documented that acetate toxicity negatively affects cell growth and ethanol production (Yang et al., 2010, 2014a), the direct underlying molecular mechanisms involved in stress responses and non-natural sugar utilization still need to be further explored. To uncover potential stress response mechanisms in response to various inhibitors in *Z. mobilis*, we focused on the discovery of regulatory RNAs using transcriptomic data and bioinformatics analysis (Cho et al., 2014). Fifteen small RNAs (sRNAs) were previously identified suggesting the potential existence of other types of regulatory non-coding RNAs in this organism, such as regulatory 5′UTRs that have not yet been annotated (Cho et al., 2014).

Regulatory RNAs include 5′ and 3′ untranslated regions (UTRs), riboswitches, *cis*-acting antisense RNAs, and *trans*-acting small non-coding RNAs that regulate gene expression, sometimes in response to external stress (Beisel and Storz, 2010; Vazquez-Anderson and Contreras, 2013; Cho et al., 2014). 5′UTRs have been reported to modify gene regulation in both prokaryotes and eukaryotes on the basis of the changes in temperature, pH, and other metabolites (Gripenland et al., 2010). For example, the 5′UTR of *prfA* mRNA immediately responds with a structural change upon temperature changes in *Listeria monocytogenes*, which is critical for survival for pathogenic bacteria in the host (Toledo-Arana et al., 2009). The 5′UTR of *alx* gene is a pH sensor in *Escherichia coli* that changes structure to allow translation of *alx* in alkaline conditions (Nechooshtan et al., 2009).

Riboswitches represent yet another class of sensors in which metabolites control gene expression in various metabolic pathways. Upon sensing small molecule metabolites, riboswitches trigger structural changes to regulate transcription or translation of mRNAs. Riboswitches consist of two components: an aptamer and an expression platform. Aptamer domains are between 35 and 200 nucleotides and responsible for direct binding to small molecule metabolites such as ions, nucleotides, amino acids, or coenzymes (Mandal and Breaker, 2004; Soukup and Soukup, 2004; Roth et al., 2007). While aptamer domains are highly structured and conserved among different species, expression platforms can vary in sequence and structure and undergo different conformational changes in response to ligand binding to aptamer domains, resulting in altered downstream gene expression, either activation or repression (Winkler et al., 2002; Winkler and Breaker, 2003; Barrick and Breaker, 2007; Vazquez-Anderson and Contreras, 2013).

Interestingly, a new class of RNA elements, OLE (ornate, long and extremophile), is highly expressed, stable, and interact with OLE-associated protein (OAP) to protect extremophiles in response to ethanol stress (Ko and Altman, 2007; Wallace et al., 2012). Although there is no evidence of OLE RNAs in *Z. mobilis*, this recent finding provided us with the hypothesis that ethanol-responsive RNA elements could be present throughout the *Z. mobilis* genome and that these could be important for its ethanol tolerance capability.

Although many different types of regulatory elements such as riboswitches and 5′UTRs have been discovered among various bacterial species, none have been experimentally confirmed in *Z. mobilis*. Three riboswitches (two cobalamine and one TPP riboswitches) have been predicted in *Z. mobilis* genome by computational analysis using CMfinder, suggesting that these regulatory elements are likely present in this organism (Yao et al., 2006). In this work, available transcriptomic data were searched with a bioinformatics approach to discover potential 5′UTRs that play a role in ethanol stress response. These were then experimentally tested for their regulatory roles and biological relevance under stress conditions utilizing an *in vivo* GFP (Green Fluorescence Protein) reporter system and classical genetic approaches.

## Results

### Identification of 101 potential 5′UTRS in *Z. mobilis* using transcriptomic data

**Figure 1:**
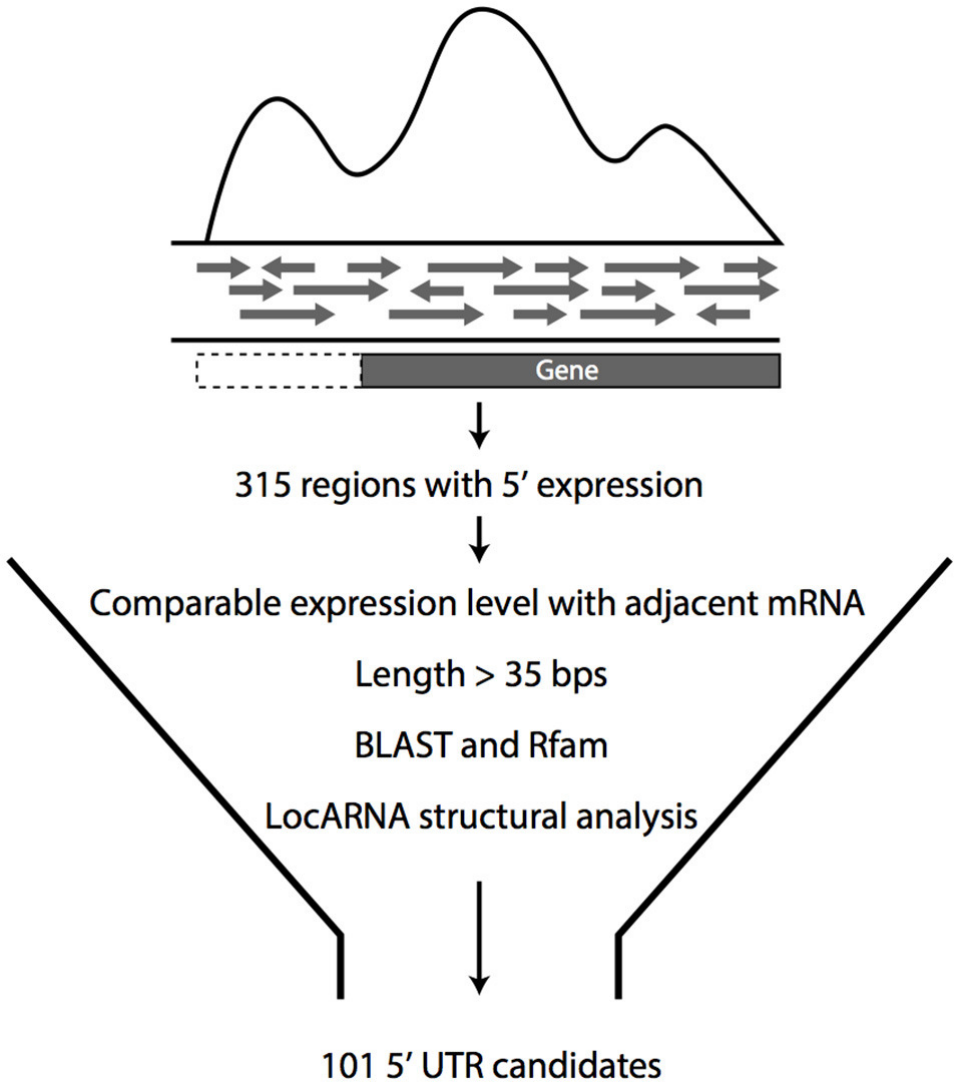
Pipeline for the selection of 5′UTR candidates. Initial 5′UTR candidates were selected from transcriptomic data and then filtered for comparable expression level to adjacent mRNA, with length more than 35 bp. 5′UTR candidates that met these criteria were analyzed for homology with known RNA regulators by Rfam and for general conservation in other organisms by BLAST. From this analysis, 101 candidates were selected for experimental confirmation.

Using previously published datasets (Cho et al., 2014), transcripts were screened for their expression in the 5′UTR regions of the adjacent coding regions to discover putative 5′UTR regions that could contribute to the gene regulation in *Z. mobilis*. Initially, 392 potential candidates were identified, leading to the need of filtering these 5′UTR candidates from the large number and diversity of other types of transcripts. For this purpose, a bioinformatics pipeline was developed to select candidates from the large number of initial candidates (Figure 1). Only candidates that showed comparable levels of expression with the adjacent genes were retained as potential UTRs. All transcripts <35 base pairs were filtered out, as this is the shortest known length of a UTR regulatory element (Roth et al., 2007). As well-characterized metabolic enzymes are highly regulated by their 5′ UTRs, these types of candidates were prioritized in the list. Collectively, this analysis resulted in a total of 101 potential candidates that were selected for experimental analysis (Table S1).

### Discerning properties of 5′UTRs through bioinformatics analysis

To further discern the properties of potential 5′UTR candidates, bioinformatics analyses were performed using Rfam (a database of multiple sequence alignments), consensus secondary structures, and covariance models representing RNA families (Griffiths-Jones et al., 2003). There are three RNA categories in Rfam: non-coding RNAs, structured *cis*-regulatory elements, and self-splicing RNAs (Nawrocki et al., 2015). Four candidates were matched with predicted riboswitches in *Z. mobilis*: TPP, crcB, and two cobalamin switches (Table 1). TPP and cobalamin riboswitches are widely conserved and have been demonstrated in *E. coli* and other bacteria, such as *Bacillus subtilis*, to control the regulation of downstream genes by direct binding to thiamine pyrophosphate and cobalamine, respectively (Nahvi et al., 2002; Winkler et al., 2002). The crcB RNA motif identified by Rfam is known as a fluoride riboswitch (Baker et al., 2012) adjacent to the 5′ end of the chloride channel protein gene (ZMO0547). This fluoride switch regulates gene expression based on its structural change in response to fluoride ions. Genes encoding this fluoride-specific type of chloride channel protein have been shown to be regulated by fluoride riboswitches in a variety of organisms (Stockbridge et al., 2012). Importantly, these results supported the presence of 5′UTRs before experimental confirmation and also validated the UTR bioinformatics prediction methods.

**Table 1:** List of 5′UTR candidates with features.

**NCBI Gene ID**	**Gene**	**Conservation by BLAST**	**Rfam prediction**	**Characteristics of known riboswitches in other organisms**	**Transcript expression under stress**	**Protein expression in ethanol stress**	**Type of stress induced differential GFP expression**
ZMO0172	Thiamin biosynthesis protein/phosphomethylpyrimidine synthase	Highly conserved	TPP	TPP riboswitch in *E.coli*	–	–	Acetate; xylose
ZMO0979	TonB-dependent receptor	Highly conserved	Cobalamin	AdoCbl	Down in acetate stress; down in xylose stress	–	–
ZMO1000	5-methyltetrahydropteroyltriglutamate	Highly conserved	Cobalamin	metE in *E.coli*/*Bacillus*	Up in xylose	–	Acetate
ZMO0547	chloride channel core	Conserved with *Gluconobacter*	*crcB*	Fluoride riboswitches	–	–	–
ZMO0056	Glucosamine/fructose-6-phosphate aminotransferase	–	–	*glmS* ribozyme	–	–	–
ZMO0376	ATP-dependent protease La	–	–	–	–	–	–
ZMO0546	sulfate transporter	–	–	ABC transporter family	Up in 5% ethanol stress; up in xylose stress	–	Acetate
ZMO0660	DnaK molecular chaperone DnaK	–	–	–	–	–	–
ZMO1069	molecular chaperone DnaJ	Highly conserved	–	–	–	–	–
ZMO1139	Acetolactate synthase large subunit	–	–	–	Up in xylose	–	–
ZMO1142	Thioredoxin reductase	–	–	–	Up in xylose stress; down in ethanol	Up	Ethanol
ZMO0709	Phosphoribosylaminoimidazole synthetase	Conserved with *Lactobacillus*	–	–	–	–	–
ZMO1137	Phosphoserine phosphatase SerB	Somewhat conserved	–	*serC* regulated by glycine riboswitch	–	–	–
ZMO0187	3-deoxy-7-phosphoheptulonate synthase	–	–	–	–	–	Xylose
ZMO0369	Glucokinase	–	–	–	–	–	–
ZMO0405	ATP-dependent Clp protease ATP-binding subunit ClpA	–	–	–	Up in xylose stress; down in ethanol stress	Up	–
ZMO0937	Aromatic amino acid aminotransferase	–	–	–	Down in acetate stress; down in xylose stress	–	–
ZMO1179	(uracil-5)-methyltransferase	–	–	–	Up in ethanol stress	–	–
ZMO1275	Threonine dehydratase	Conserved with *Clostridium*	–	–	–	–	–
ZMO0689	Oxidoreductase domain-containing protein	–	–	–	Down in xylose stress	Up	–
ZMO0131	Metallophosphoesterase	–	–	–	Down in acetate stress; down in xylose stress	–	–
ZMO0748	Cysteine synthase	–	–	–	Up in acetate stress; up in xylose stress	–	–
ZMO1034	Calcium-binding EF-hand-containing protein	–	–	–	Up in acetate stress; up in xylose stress	Up	–
ZMO1113	FAD-dependent pyridine nucleotide-disulfide oxidoreductase	–	–	–	Up in xylose	–	–
ZMO0347	RNA-binding protein Hfq	Conserved with *Clostridium*	–	–	Down in ethanol stress	Up	Ethanol
ZMO1198	5-aminolevulinate synthase	–	–	–	Down in xylose stress	–	–
ZMO1478	6-phosphogluconolactonase	–	–	–	–	–	Acetate
ZMO0275	ABC transporter	–	–	–	–	–	–
ZMO1399	fatty acid hydroxylase	–	–	–	–	–	–
ZMO1432	Fusaric acid resistance protein	–	–	–	–	–	–
ZMO0367	glucose-6-phosphate 1-dehydrogenase	–	–	–	Up in ethanol stress	Down	–
ZMO1412	MucR family transcriptional regulator	–	–	–	Down in ethanol stress	Up	–
ZMO1048	Phosphate ABC transporter inner membrane subunit PstC	–	–	–	–	–	–
ZMO0140	Protein tyrosine phosphatase	–	–	–	–	–	–
ZMO0366	Sugar transporter	–	–	–	–	–	–
ZMO1612	Toluene tolerance family protein	–	–	–	–	–	–

Given that structural conservation is closely associated with the regulatory roles of RNA (Yang et al., 2010), LocARNA was used to determine conservation based on both sequence and structure (Will et al., 2012). NCBI BLAST (Camacho et al., 2009) was used to identify sequence homology for each 5′UTR candidate, as required for LocARNA input. Of the initial 101 5′UTR candidates, 28 contained structurally conserved motifs, many of which showed complexity. Figure S1 shows representative data of this analysis.

Lastly, differential expression data under ethanol, acetate, and xylose stresses were compiled for the genes associated with the 5′UTR candidates (He et al., 2012a; Yang et al., 2013, 2014a,b). Genes with differential expression under one or more stresses may be regulated by a 5′UTR or other mechanism. As shown in Table 1 and Table S3, 17 mRNAs and 7 proteins corresponding to the 5′UTR candidates were up- or down- regulated under stress. Due to their association with stress response(s), these candidates were further investigated in this work.

### Validation of 36 5′UTR candidates by RT-PCR analysis

**Figure 2:**
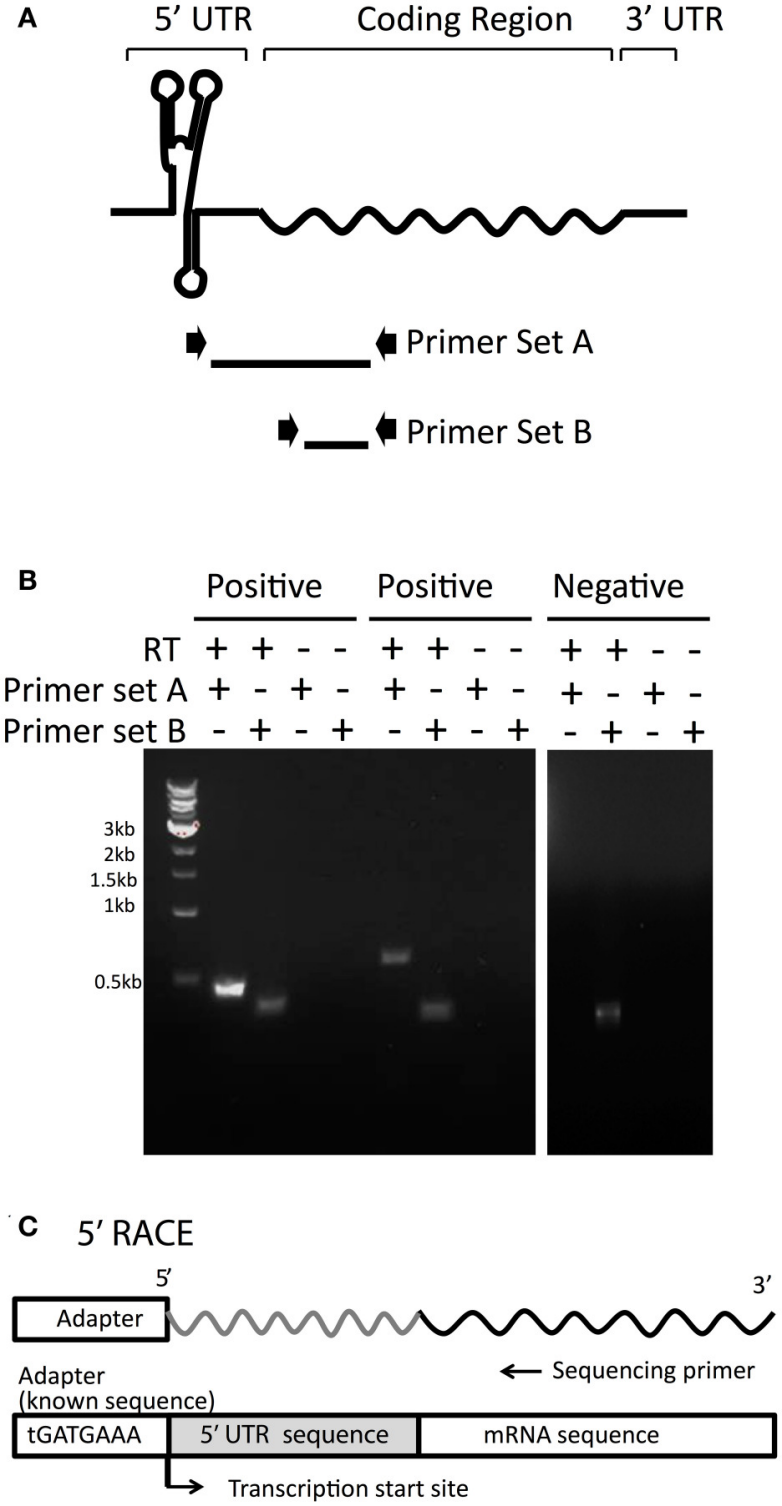
RT-PCR analysis to experimentally confirm 5′UTR candidates. **(A)** To confirm the 5′UTRs detected in transcriptomic data are actually transcribed with their downstream mRNAs, two sets of primers were designed. Primer Set A amplifies from the middle of the predicted UTR into the adjacent mRNA coding region. Primer Set B, as a control, amplifies the coding region only, indicating the amplification level of the transcript. **(B)** Examples of positive (confirmed) and negative (undetected) 5′UTR candidates are shown. RNA was reverse-transcribed and then subjected to PCR with Primer Set A and Primer Set B for each 5′UTR candidate. As a negative control, RNA was left without reverse transcription to indicate background levels of any residual DNA that could be left undigested from DNaseI treatment. Samples were then visualized on agarose gels, as shown in the examples. **(C)** For each 5′UTR confirmed by RT-PCR, 5′RACE was performed to determine the transcription start site by attaching an adapter of known sequence to the 5′ end and sequencing from the mRNA coding region upstream toward the adapter.

The cellular expression of all 101 final candidates (Table S1) was further confirmed by RT-PCR analysis, for which two primer sets were designed (Figure 2A). Primer set A was designed for the amplification of a long transcript from hypothetical 5′UTR regions to the middle of the corresponding mRNA coding region. Primer set B was designed to amplify a relatively short transcript inside the adjacent mRNA coding region as a positive control representing the adjacent mRNA expression level (Figure 2A). As a negative control, reverse transcriptase was excluded from the reaction to confirm the absence of genomic DNA contamination. Representative data are illustrated in Figure 2B and all RT-PCR results are shown in Figure S2. PCR bands from both primer sets A and B proved the expression of transcript containing potential UTRs upstream. However, the presence of a band from primer set B and not from set A, indicated that the UTR was not detected upstream of the mRNA. From the experimental analysis, fifty 5′UTR candidates that showed contiguous expression in the transcript along with the mRNA, which are marked as confirmed candidates in Table S1. Lack of detectable expression of other candidates could be explained by unsuccessful PCRs, false positives in our candidate selection from the transcriptomic data, or different growth conditions between RT-PCR and previous transcriptomic studies.

Precise transcription start sites (TSS) for all candidates of interest were determined by 5′ RACE (Figure 2C). A summary of the 36 final candidates that were further investigated and their gene function is included in Table 1 and additional information in Table S2. It should be noted that 14 candidates were excluded from further analysis out of the 50 experimentally confirmed candidates due to their overlapping transcription with the adjacent gene (3′ end of the adjacent gene was connected with potential 5′ UTRs), length <30 bp, or lack of experimental validation by RT-PCR and/or 5′RACE (Figure S3).

### Establishment and validation of the high-throughput fluorescence-based screening system

**Figure 3:**
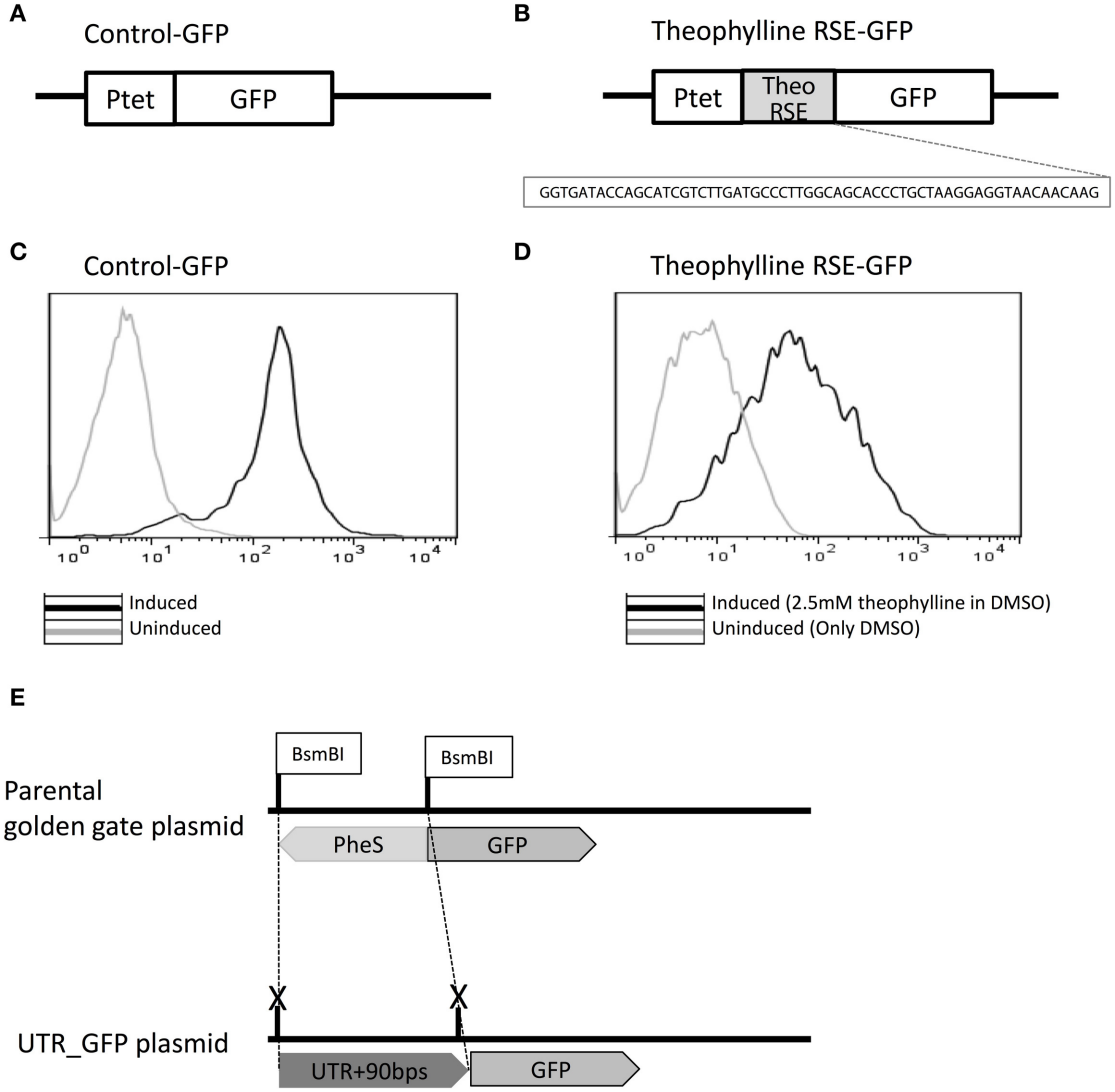
Establishment of a green fluorescent protein (GFP)-based high-throughput reporter gene screening system to characterize regulatory 5′UTR regions. Control-GFP plasmid was developed in which GFP is located under P_tet_ promoter control to demonstrate expression of the GFP protein in *Z. mobilis* **(A)**. The theophylline synthetic riboswitch (sequence shown) was cloned in front of GFP to regulate its expression and show that a well-characterized regulatory 5′UTR could be used to see measurable differences in GFP expression upon the addition of the ligand it senses (theophylline in this case) **(B)**. Upon induction of P_tet_ with tetracycline (black), the Control-GFP strain shows a fluorescence shift by flow cytometry compared to the un-induced sample (light gray) **(C)**. The strain containing plasmid theophylline RNA stability element (RSE)-GFP was induced with 2.5 mM theophylline in DMSO (black) and compared by flow cytometry to a sample to which only DMSO (light gray) was added. In both cases, P_tet_ was induced with tetracycline **(D)**. To allow screening of 5′UTRs identified in this study, the plasmid was designed with BsmBI sites flanking the PheS cassette in front of GFP. The PheS cassette was replaced with each UTR+90 bps of initial mRNA sequence by Golden Gate cloning **(E)**.

To test each 5′UTR candidate's ability to regulate downstream gene expression, an *in vivo* fluorescence-based reporter gene screening system was developed, taking advantage of previous successful efficient expression of GFP in *Z. mobilis* (Douka et al., 2001). As shown in Figure 3A, inducible expression of GFP was confirmed under the P_tet_ promoter, and then the 60-nucleotide sequence of the theophylline riboswitch element was cloned in front of the GFP gene to establish the functionality of the GFP screen in *Z. mobilis* (Figure 3B). **T**he fluorescence shifted when it is induced with 10 μg/mL tetracycline compared to that of the un-induced control sample (Figure 3C). After confirming the functionality of the fluorescence system in *Z. mobilis*, the well-characterized theophylline synthetic riboswitch was used as a test case to establish the screening system that was able to elicit a fluorescence change specific to the activation of a 5′UTR. The theophylline riboswitch has been engineered as a synthetic riboswitch system to control gene expression in various bacterial species and natively controls gene expression at the translational level by binding to the small molecule, theophylline (Suess et al., 2004; Lynch et al., 2007; Topp et al., 2010).

To establish the functionality of the GFP screen, the 60-nucleotide sequence of the theophylline riboswitch element was cloned in front of the *gfp* gene in *Z. mobilis* (Figure 3B) and the level of GFP expression with 2 mM theophylline was compared with a DMSO control in *Z. mobilis*. Control-GFP exhibited about 10-fold GFP expression when it was induced (Figures 3C,**D**). In contrast to control-GFP, a 2-fold increase in GFP expression from theophylline RNA stability element (RSE)-GFP construct was observed. Although the fold change in GFP was not high (limited to about 2-fold), this was enough to screen for positively activating candidates. It is also worth noting that this is not an unusual fold change of expression when using the same theophylline riboswitch in different bacteria species (Topp et al., 2010) without engineering pairing strength of the region between the aptamer and RBS (Ribosome Binding Site) in the theophylline switch within a particular species. Importantly, the activation of the theophylline switch was successfully demonstrated, as well as the use of this 5′UTR *in vivo* fluorescence-based screening system in *Z. mobilis* for the first time. This could be a useful tool for screening the control of gene expression in metabolic pathways related with ethanol tolerance or other stress responses in *Z. mobilis*.

### Identification and characterization of stress-responsive regulatory 5′UTRs using reporter gene system

Utilizing the *Z. mobilis* pEZ minimal shuttle vector system (Yang et al., 2016b), a high-throughput cloning strategy was developed for construction of each 5′UTR-containing GFP plasmid by the combination of Golden Gate assembly and PheS counter selection marker (Figure 3E). Given that nucleotides in the coding region may affect the structure of 5′UTRs for the regulation of the gene, each 5′UTR sequence (verified by 5′ RACE) was cloned with an additional 90 base pairs of the downstream coding region of the corresponding gene for the generation of 5′UTR-GFP libraries. Primers used for the generation of 5′UTR-GFP libraries are listed in Table S3. Using this plasmid construct, a library of 36 5′UTR-GFP candidates was generated (Table 1) for further testing. The sequences and features of the final 36 candidates were shown in Table S2.

Since high tolerance to ethanol is one of the desirable features of *Z. mobilis*, the initial screen of 5′UTR-GFP libraries was conducted under ethanol stress (5% (v/v) ethanol-supplemented media) to evaluate potential 5′UTR activation relative to a standard medium (RM) control. A strain with only GFP and no 5′UTR (Control-GFP) was used as a negative control. All experiments were done in biological triplicates. After the signal difference was observed to be highest at 10 h post-induction, the fluorescence measurements were taken at this time point for all other experiments. This is consistent with previous data that showed maximum GFP fluorescence in late exponential phase (Douka et al., 2001). Upon screening all candidates, two were identified that exhibited significant fluorescence changes under 5% ethanol supplementation compared to the control without added ethanol: the 5′UTR of ZMO0347 (RNA binding protein Hfq, UTR_ZMO0347) and the 5′UTR of ZMO1142 (thioredoxin reductase, UTR_ZMO1142) (Figure S4).

**Figure 4:**
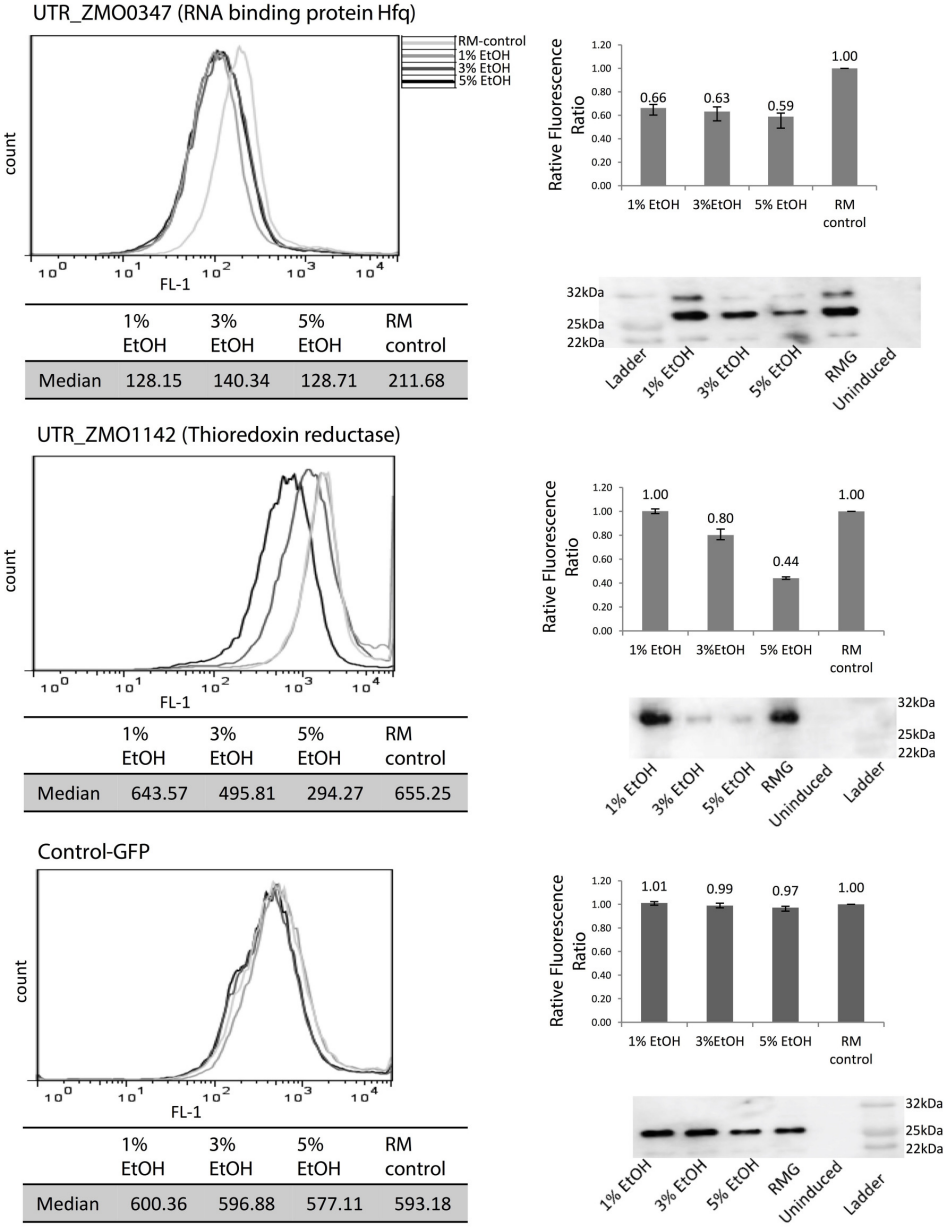
Identification of 5′UTRs that regulate GFP expression under ethanol stress. UTR_ZMO0347 and UTR_ZMO1142 showed fluorescence shifts under different levels of ethanol compared to control-GFP. White gray line: RMG (normal media). Light gray line: 1% ethanol supplemented media. Dark gray line: 3% ethanol supplemented media. Black line: 5% ethanol supplemented media. Median fluorescence level of each condition is shown in a table and the fluorescence ratios relative to the RMG control are shown in the bar graphs. GFP protein levels were detected by western blotting using anti-GFP antibody. These experiments were done in triplicate and the fluorescence ratio bar graph error bars represent standard deviation from the mean.

To explore responsiveness of these 5′UTRs under different levels of ethanol, 1, 3, or 5% (v/v) of ethanol were added to the cultures before fluorescence and western blotting analysis (Figure 4). In this plasmid system, GFP fluorescence uniformly decreased under any level of ethanol stress when regulated by the UTR_ZMO0347 (RNA binding protein Hfq), whereas the fluorescence under the control of UTR_ZMO1142 (thioredoxin reductase) showed an ethanol concentration-dependent decrease (Figure 4). The changes in GFP expression in cells were also confirmed by western blotting analysis, which corresponded well with fluorescence data (Figure 4). All 5′UTR candidates were also tested for responses to xylose and acetate stresses in this fluorescence screening system (Figure S5). Interestingly, UTR_ZMO1142 did not appear to regulate gene expression under xylose and acetate stresses, but only under ethanol stress (Figure S5). Consistent with these results, it has been reported that thioredoxin reductase (ZMO1142) is less abundant under 6% ethanol stress at the protein level, although not at the transcript level, indicating a layer of translational regulation (Yang et al., 2013). Additionally, for Hfq (ZMO0347), a protein that is highly associated with stress response in various organisms, a decrease of transcripts has been previously detected under 6% ethanol stress (Guisbert et al., 2007; Yang et al., 2013; Torres-Quesada et al., 2014). However, inconclusive data about changes in Hfq protein levels are found throughout the literature (and our own studies) given the difficulty in detecting this protein by mass spectrometry. Importantly, the responses of UTR_ZMO0347 and UTR_ZMO1142 to ethanol indicate that UTR regulation could be part of a mechanism of ethanol stress protection in *Z. mobilis*.

In addition to ethanol stress responsive 5′UTRs, a few acetate and xylose responsive elements were identified (Figure S5). A previous study confirmed that regulatory mechanisms responding to acetate stress and xylose utilization mainly included differential expression of carbon and energy metabolism genes to reduce the impact of stress on the cell (Yang et al., 2014a). Acetate stress repressed genes associated with flagellar system and glycolysis, but induced genes related to stress responses and energy metabolism (Yang et al., 2014b). 6-phsphogluconolactonase catalyzes hydrolysis of the ester linkage of lactone, resulting in production of 6-phophogluconate in the pentose phosphate pathway. Up-regulation of ZMO1478 (6-phosphogluconolactonase) transcript in acetate stress has been previously reported (Yang et al., 2014a), and in this study, UTR_ZMO1478 (6-phosphogluconolactonase) was shown to activate GFP expression under acetate stress (10 g/L sodium acetate). Taken together, these results suggest that 5′UTR could be responsible for the natural increase of ZMO1478 expression under acetate stress. Additionally, UTR_ZMO0172 (thiamine biosynthesis protein) showed down-regulation of GFP expression under both acetate and xylose stresses, indicating that this gene could be involved in general stress response mechanisms. Further studies on protein expression levels of ZMO0172 under stressful conditions could help reveal the underlying regulatory mechanism of UTR_ZMO0172.

### Native regulation of Hfq translation by 5′UTR

To evaluate the physiological effects of ethanol stress-responsive regulatory 5′UTRs in *Z. mobilis*, a mutant strain was constructed with a genomic deletion of UTR_ZMO0347. Unfortunately, UTR_ZMO1142 could not be deleted due to the limitation of the technique used in this study for short length deletion (e.g., 57 bp in this study for UTR_ZMO1142). The UTR_ZMO0347 deletion was constructed utilizing a homologous recombination deletion technique to disrupt the 5′UTR region (except for promoter and RBS regions) of the *hfq* gene in *Z. mobilis* 8b strain. The spectinomycin resistance gene was used as a selection marker between 1 kb up- and down-stream homology arms. The deletion of this region was confirmed by PCR and Sanger sequencing (data not shown). Primers used for the construction and confirmation of deletion constructs are shown in Table S3. Growth data for the *Z. mobilis* parental strain 8b and its *hfq* 5′UTR deletion mutant strain were collected (Figure S6). A growth defect in the ΔUTR_ZMO0347 strain was observed relative to the wild type strain under no ethanol stress.

**Figure 5:**
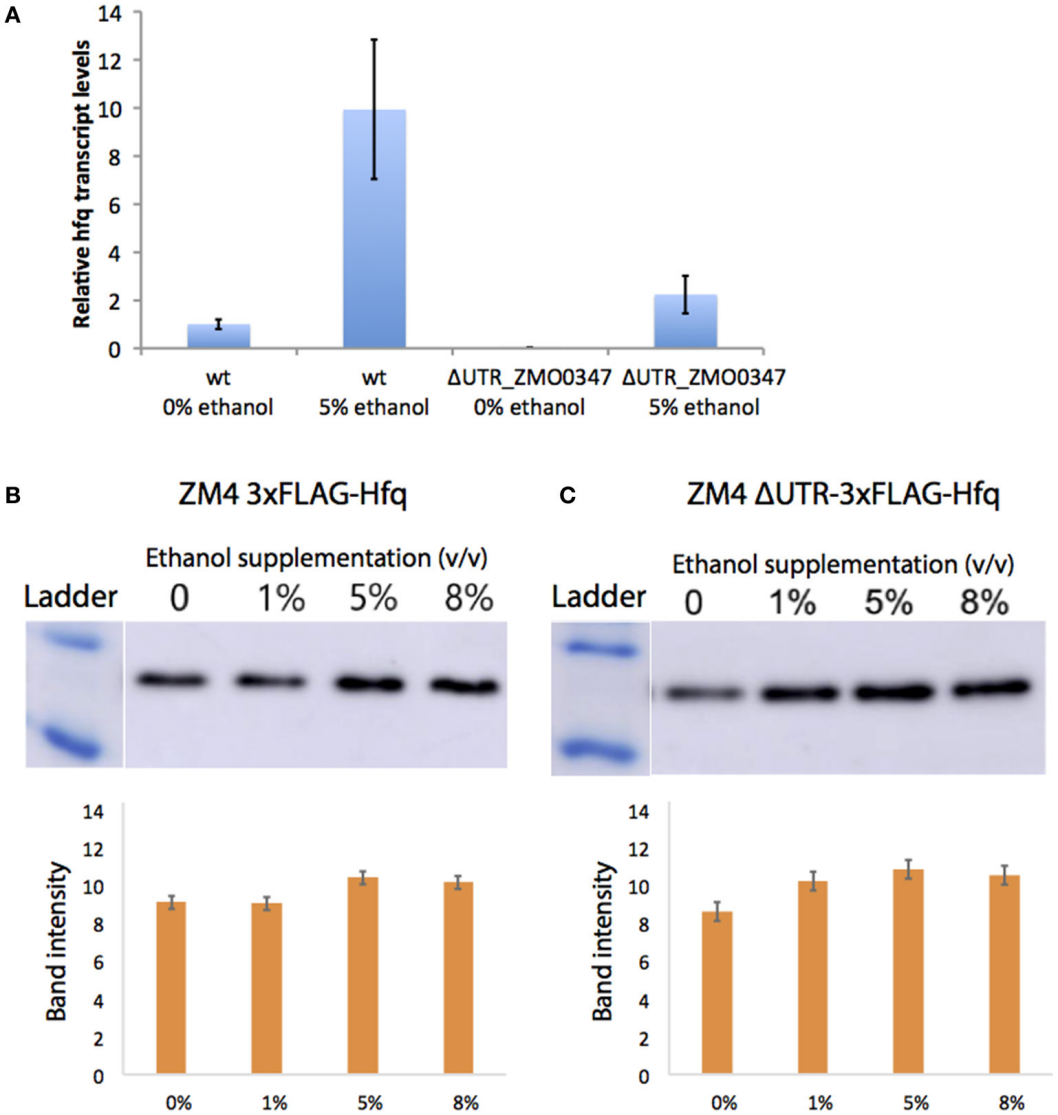
Native regulation of Hfq in ethanol stress. Transcript levels of *hfq* were quantified by qPCR with (5%) and without (0%) ethanol supplemented to the media **(A)**. Bars represent averages of biological triplicates calculated by the comparative delta-delta threshold cycle (ΔΔC_T_) method and error is standard deviation. The Hfq protein levels were quantified by western blots of chromosomally FLAG-tagged Hfq strains with the UTR **(B)** and without **(C)**. After growth in a range of ethanol concentrations, 20 ng of total protein were loaded into an SDS-PAGE gel, then transferred to a membrane, and blotted with the anti-FLAG antibody. Band intensity was quantified and is shown as averages of biological triplicates with error as standard deviation.

Transcript levels of the ZMO0347 (Hfq) mRNA in the deletion strain were measured via qRT-PCR with and without 5% (v/v) ethanol supplementation (Figure 5A). Although the deletion strain shows an overall decrease in transcription, a consistent up-regulation of the *hfq* transcript is observed in both the wild type and deletion mutant strains in the presence of ethanol. This suggests that the ethanol-responsive regulatory effect of the 5′UTR is not at the transcriptional level (e.g., due to promoter effects).

At the protein level, native Hfq levels were also evaluated in the ΔUTR_ZMO0347 mutant strain relative to its wild-type ZM4 strain via western blotting analysis (in chromosomally FLAG-tagged Hfq strains for both the wild type ZM4 and the ΔUTR_ZMO0347 strain). As shown in Figures 5B,C for the wild type and deletion strains respectively, the Hfq protein showed a small (less than a 2-fold increase) change in response to ethanol. In the wild type strain, increases in Hfq protein expression were observed starting with 5% (v/v) ethanol supplementation (but not with 1% ethanol supplementation). In contrast, in the ΔUTR_ZMO0347 deletion strain, a more sensitive response was observed upon supplementation with ethanol, in which the increase in Hfq protein was observed with only 1% (v/v) ethanol supplementation (Figure 5). This suggests that the native 5′UTR region serves as a post-transcriptional regulator, akin to a resistor, that keeps Hfq protein levels down-regulated in response to lower ethanol stress. Given the fact that the isolation of 5′UTRs in our GFP assays (in the absence of their native promoters and genetic context) indicated down-regulation of protein expression by the 5′UTR, the overall increase of *hfq* transcript and protein levels in the wild type strain with ethanol suggests that additional regulatory effects play a role in overall Hfq regulation. As discussed below, this is not surprising given that, in *E. coli* and other organisms, Hfq is globally regulated by highly complex schemes that involve its native promoter and global protein-sRNA regulators (Vecerek et al., 2005; Sobrero and Valverde, 2012; Ramos et al., 2014; Sowa et al., 2017).

## Discussion

Bacterial 5′UTR elements contribute to comprehensive gene regulation under stress conditions. They rapidly sense and respond to environmental changes in order to orchestrate a cascade of gene expression and protein activity changes (Oliva et al., 2015). Conventionally, sequence-based conservation analysis such as Rfam has been widely used to identify UTRs. However, since it is based on alignments of UTRs across organisms, this kind of approach is limited to known UTRs and to the identification of functional homologs from closely related species. Therefore, as in this study, using high-throughput transcriptomic data to identify novel 5′UTRs and functional homologs of known 5′UTRs in less-studied and non-model bacteria could significantly improve the identification of 5′UTRs and to screen the functioning of these 5′UTRs under a variety of environmental (e.g., temperature and pH) or intracellular conditions (i.e., changes in particular small molecule concentration). This work thus highlights the feasibility of using transcriptomic data, bioinformatics analysis, and fluorescence-based screening to identify novel regulatory 5′UTRs in other microorganisms.

Additionally, this study has demonstrated that 5′UTR elements in *Z. mobilis* have regulatory roles, particularly under ethanol stress conditions. UTR_ZMO0347 (RNA binding protein Hfq) and UTR_ZMO1142 (thioredoxin reductase), which are associated with ethanol stress responses, showed down-regulation of the downstream genes. Interestingly, this regulatory effect was observed more drastically under higher concentrations of supplemental ethanol for UTR_ZMO1142. The downstream gene of UTR_ZMO0347 encodes a homolog of RNA binding protein Hfq, an RNA chaperone and regulator of the small RNA network. Hfq also mediates transcription anti-termination via binding to Rho factor for the control of gene expression at the transcriptional level in *E. coli* (Rabhi et al., 2011). Previous studies also indicated that this Hfq homolog in *Z. mobilis* is associated with stress responses (Yang et al., 2009). The data in this study thus suggest that UTR_ZMO0347 mediates regulatory effects on its downstream gene *hfq*, even though it is not clear that this effect is induced by direct interaction with ethanol or through the participation of other factors involved in the stress response. Thioredoxin reductase (ZMO1142) catalyzes the reduction of thioredoxin coupled with NADPH and as such plays a major role in the defense mechanism for the oxidative stress via the reduction of disulfide bonds by thioredoxin reductase (Koháryová and Kolárová, 2008). UTR_ZMO1142 also responded to xylose and acetate stresses, implicating it as a potential regulator of the general stress response.

Because of the response of UTR_ZMO0347 (Hfq) to ethanol stress, this UTR was genetically deleted. Two major phenotypic differences were observed between the UTR mutant and the wild type strains: (1) a general decrease in *hfq* expression at the transcript level, and (2) up-regulation of Hfq protein expression within a wider range of ethanol stress levels specifically at lower levels (Figure 5). In addition to the translational regulation, this work also demonstrates that the presence of the 5′UTR affects transcriptional levels of *hfq*, likely by providing stability to its transcript. In *Staphylococcus aureus*, Hfq protein was identified as a global regulator involved in stress and pathogenicity (Liu et al., 2010), and it is suspected that this protein could have a similar role throughout bacteria. As an important regulatory factor upon stress, it has been reported that Hfq was also involved in osmotic and ethanol stress in *L. monocytogenes* (Christiansen et al., 2004), where the transcription of *hfq* was induced upon ethanol and osmotic stress depending on sigma factor σ^B^ for the regulation of stress responses in *L. monocytogenes* (Christiansen et al., 2004). However, regulation of *hfq* itself and its UTR still needs to be elucidated, particularly in response to various stresses in different growth phases in *Z. mobilis*.

**Figure 6:**
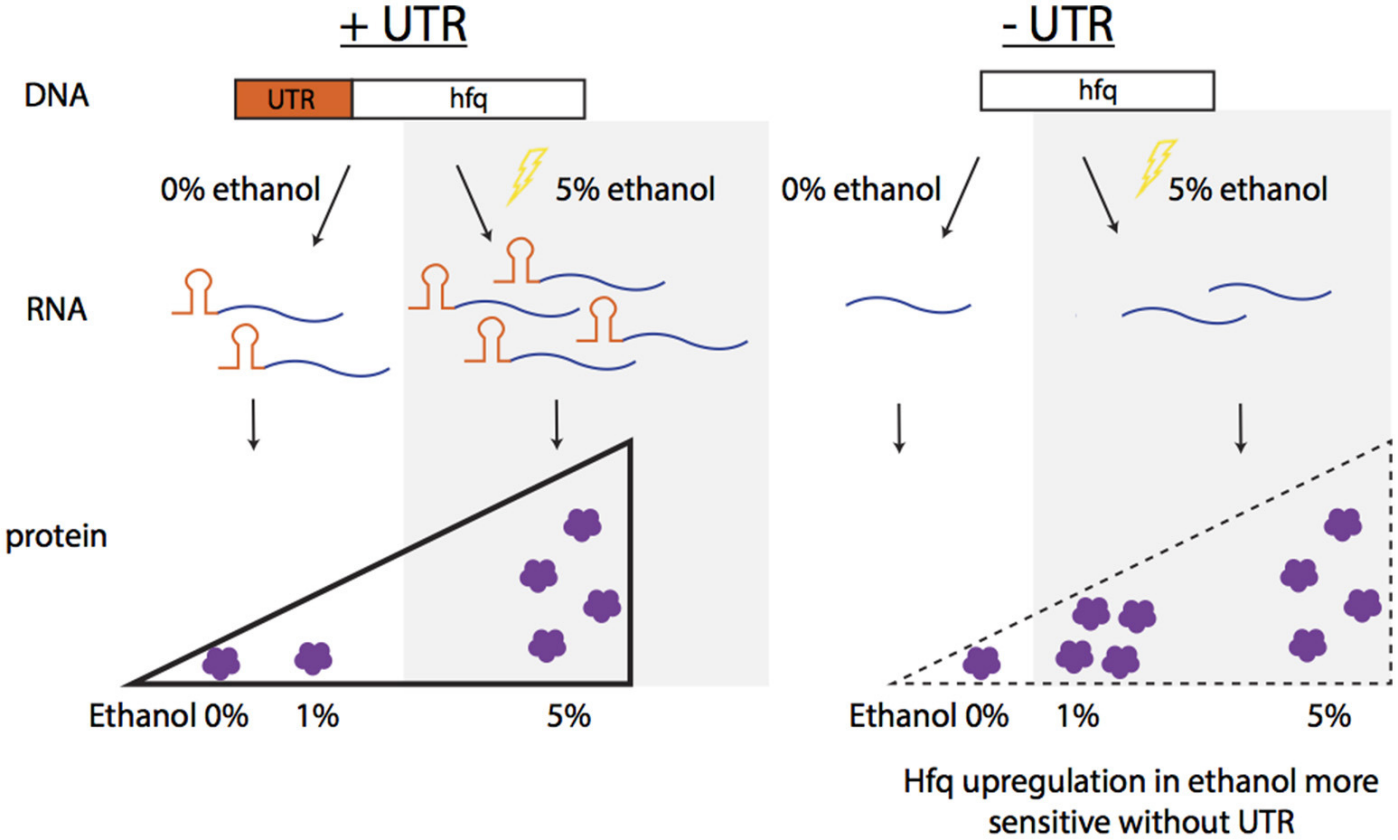
Summary of observed Hfq regulation in ethanol stress. In native conditions with the UTR of Hfq intact (ZMO0347), the transcript and protein levels increase in 5% ethanol stress. When the UTR is deleted from the genome, the protein expression increases upon a more mild ethanol stress (1%), indicating the sensitively of Hfq expression without the UTR.

Interestingly, in this work, the opposite trend of the effect on the native 5′UTR on expression of the *hfq* transcript and (mildly) on the Hfq protein was observed (increase expression in presence of 5′UTR) relative to the effect observed at the protein level with our heterologous reporter, where GFP consistently decreased when regulated by the isolated 5′UTR. This observation can be attributed to the complexity of regulatory mechanisms known to affect *hfq*. A potential model of the contribution of the 5′UTR for native Hfq regulation is illustrated in Figure 6. With the 5′UTR intact, transcript levels and the protein produced are higher with 5% ethanol supplementation than that without external addition of ethanol. Without the 5′UTR, the transcripts levels are lower, but show the same trend as with the UTR intact. Interestingly, the protein is upregulated at a lower ethanol concentration (1%) when the UTR is missing compared to when it is present (upregulation not seen at 1%, but at 5%) (Figure 5). This suggests that the Hfq protein levels are more sensitive to upregulation without the 5′UTR. The UTR′s post-transcriptional regulation role may be to minimize over-production of Hfq protein from the more abundant transcripts in mild ethanol stress. Additionally, although the transcripts are fewer in the mutant strain with the Hfq 5′UTR deleted, protein production is about the same in both strains at 5% ethanol, suggesting post-transcriptional regulation by the 5′UTR.

Overall, this study provided a strategy to identify regulatory 5′UTR using omics datasets and to characterize them using a fluorescence reporter gene system for bacterial species. It also shed a light on the regulatory role of 5′UTR regions in stress responses in *Z. mobilis* for the first time, after constructing and validating a novel genome-wide 5′UTR screening tool in this organism. A regulatory 5′UTR region on *hfq* has been identified and the regulation of Hfq in particular at the protein level appeared to be important in viability under ethanol stress for *Z. mobilis*, even though complex regulatory networks around the *hfq* gene should be further investigated. This work also provided novel responsive regulatory 5′UTR biological parts for future metabolic engineering efforts to enhance microbial robustness and temporal gene expression.

## Materials and methods

### Bacterial strain and culture conditions

*Z. mobilis* 8b was provided by NREL (Zhang et al., 1995). Cells were cultured in 5 mL RMG (glucose, 20.0 g/L; yeast extract, 10.0 g/L; KH_2_PO_4_, 2.0 g/L; pH 6.0) (Yang et al., 2009) overnight at 33°C and then inoculated into 100 mL fresh medium. Initial OD_600nm_ was ~0.05 and cells were induced with 10 μg/mL of tetracycline when OD_600nm_ reached ~0.4. 1, 3, 5, or 8% (v/v) ethanol was supplemented into RMG for ethanol stress experiments from the beginning of growth. 10 g/L of sodium acetate (NaAc) (Yang et al., 2014b) was supplemented into RMG for acetate stress. In the case of xylose stress, 10 g/L glucose + 10 g/L xylose was used instead of 20 g/L glucose in the media. Cells were collected 4 or 12 h post-induction for measuring GFP expression.

### Construction of *hfq* mutant strains

Upstream and downstream fragments (each about 1 kb) homologous to the target deletion gene were assembled with the spectinomycin gene *aadA* in the middle. The upstream homologous arm was PCR amplified using primers Fup and Rup, and the downstream homologous arm amplified by Fhfq-1, Fhfq-2, and Rhfq. Primers Fsp, Rsp-1, and Rsp-2 were used to introduce a terminator at the end of the spectinomycin resistance gene and primers Fhfq-1 and Fhfq-2 were used to introduce the 3xFLAG. The fragments were then assembled similarly to Gibson assembly. Briefly, primers were designed with 15-nt overhangs of desired upstream or downstream sequences and used to amplify the pieces for the assembly and purified from the primer dimers by gel electrophoresis followed by gel purification. Fragments and the pUC57 vector were mixed in a molar ratio of 3:1 (about 120 ng total DNA) and added to 0.5 U T5 exonuclease (NEB, USA), 0.5 μL Buffer 4 (NEB, USA), and nuclease-free water (up to 5 μL). The reaction mix was kept on ice for 5 min and then transformed into *E. coli* competent cells by chemical transformation. Cells were plated on LB agar plates with 200 μg/mL of spectinomycin. Transformants were screened by colony PCR and confirmed by Sanger sequencing (Sangon, China). The purified plasmid was electroporated into the *Z. mobilis*. Transformants appearing on RM agar plates with 200 μg/mL of spectinomycin were cultured and screened by colony PCR. Colonies with correct PCR product sizes were selected as deletion candidates after Sanger sequencing confirmation.

### 5′ rapid amplification of cDNA ends (RACE)

RACE experiments were performed on total RNA samples using FirstChoice® RLM-RACE kit (Ambion, CA) according to the manufacturer′s protocol and to previously published work (Cho et al., 2014). Briefly, 8 μg RNA was treated with Tobacco Acid Pyrophospatase (TAP) at 37°C for 1 h, followed by ligation of the 5′ RACE kit adapter at 37°C for 1 h. The resulting RNA was then reverse transcribed according to the manufacturer′s protocol and PCR was performed on the resulting cDNA. All primer sequences used for RACE are listed in Table S3. Resulting PCR products were purified using QIAquick PCR purification kit (Qiagen, MD) and RNase-free water (Ambion, CA) for final elution. Final PCR products were sequenced and results were compared with the genome. 5′ RACE adapter sequences (5′-GCUGAUGGCGAUGAAUGAACACUGCGUUUGCUGGCUUUGAUGAAA-3′) and adjacent mRNA sequences were used for the detection of TSS in the 5′UTRs.

### Construction of GFP-reporter plasmids with 5′UTRs

The tetracycline-inducible promoter with GFP construct (pEZ-tet-GFP) was derived from the minimal shuttle vector pEZ15Asp of *Z. mobilis* and contains the replication origin of *E. coli* and *Z. mobilis* (Yang et al., 2016b). A parental plasmid containing *pheS* counter-selection marker (Kast, 1994; Miyazaki, 2015) was constructed with *pheS* counter-selection marker incorporated in front of the *gfp* gene flanked by BsmBI sites (Type IIS enzyme), which is one of the Type IIS restriction endonuclease to enable Golden Gate cloning for efficient cloning of each 5′UTR-containing GFP (Engler et al., 2008; Figure 3E). With this design, 5′UTRs along with the first 90-bps of the downstream mRNA were cloned for each candidate right in front of the *gfp* gene in frame. This design was implemented to preserve the native structure of the UTR by better mimicking its native context while still allowing GFP expression. Primers used for the amplification of UTR+90-bps are listed in Table S3. Each primer contains a BsmBI enzyme site on the 5′ end.

### Fluorescence measurements

Cells were analyzed by flow cytometry using the FACSCalibur™ (BD Biosciences, CA) according to standard methods as described in a previous study (Miyazaki, 2015). Briefly, cells were pelleted and resuspended in phosphate buffered saline (PBS: 137 mM sodium chloride, 2.7 mM potassium chloride, and 10 mM phosphate buffer, pH 7.5) to a concentration on the order of 10^7^ cells/mL. The cells were excited with the 488 nm argon laser and the cell population was determined from the forward scatter and side scatter distributions reported by the cytometer. Data were collected for at least 50,000 active cells, ensuring enough events to assume that the population distribution would be unaffected by rare events. Sample data were analyzed using CellQuest Pro (BD Biosciences) with a user-defined gate. Averages of median values for each sample were calculated from biological triplicates and error bars were calculated as standard error of the mean.

### Quantitative RT-PCR

RT-PCR was used to experimentally confirm the presence of 5′UTRs from the candidate list (Table S1). Total RNA was prepared using Trizol reagent (Invitrogen, CA) and resulting RNA was treated with DNase I (RNase free, ThermoScientific, MA) to prevent of genomic DNA contamination as described in the manufacturer′s protocol. Subsequently, RNA was precipitated with isopropanol and then washed with ethanol. Total RNA rehydrated with nuclease-free water (Ambion, CA) was used as the template for reverse transcription. Two hundred nanograms of RNA was incubated for the first strand synthesis with 100 ng of random hexamer and 10 mM dNTPs at 65°C for 5 min. According to the manufacturer′s protocol, SuperScript™ III Reverse Transcriptase (Invitrogen, CA) was added to RNA-primer mix with RNaseOUT™ RNase Inhibitor, 0.1 M DTT, 5x First-strand buffer and then incubated for 5 min at room temperature. The final reaction mixture was incubated at 55°C for 1 h and then heat inactivated at 70°C for 15 min.

The cDNA product from first-strand synthesis was used as a template in a 20 μL PCR reaction containing Power SYBR® Green PCR master mix (Invitrogen, CA). Specific primers were used for each target. For example, primers F (5′- GCGTCTTGTTGACCCGTAAT−3′) and R (5′- AATCCTCGTCTCGCCTTTCT−3′) were used for the amplification of the *hfq* gene. Three primers were designed for 5′UTR RT-PCR: the first forward primer (with reverse primer, set A) was located in the middle of the 5′UTRs and the second forward primer (with reverse primer, set B) was designed to bind in the front part of following mRNA regions. The reverse primer was designed to bind in the middle of the mRNA regions. All primers used in this study are listed in Table S3. Phusion® High-Fidelity DNA Polymerase (NEB, MA) was used for PCR amplification. No reverse transcriptase was used for the negative control to exclude potential genomic DNA contamination. Primer set B was used for the positive control as it represents amplification of the mRNA coding region. The temperature cycle used for the PCR reactions is as follows: 95°C for 10 min, 40 cycles of 95°C for 15 s and 60°C for 60 s. Relative quantification of *hfq* transcript between each condition was performed using Viia 7 Software (Life Technologies, CA) following the comparative delta-delta threshold cycle (ΔΔC_T_) method. Samples were collected in biological triplicates.

### Western blotting analysis and quantification of protein expression levels

Western blotting analysis was performed to detect GFP expression using anti-GFP antibody (Roche 11814460001). Standard western blotting protocols were used as previously described (Cho et al., 2016). Briefly, total cellular lysates were loaded onto a 12% denaturing SDS-PAGE gel. Gels were transferred to methanol activated PVDF membranes using the Trans-Blot® Semi-Dry Electrophoretic Transfer Cell (Bio-Rad, CA) and run for 40 min at 15 V or 20 min at 25 V. Membranes were blocked with 5% dry non-fat milk in Tris-buffered saline (TBS, pH 7.5) for 1 h at room temperature. The proteins were detected with anti-GFP antibody at 1:1000 dilutions or anti-FLAG at 1:5000 (Proteintech, China). As a secondary antibody, goat anti-mouse IgG (H + L) HRP Conjugate (Promega #W4021) was used at a dilution of 1:2500 (or 1:5000 with Proteintech, China). All images were developed using Clarity™ ECL Western Blotting Substrate (BioRad, #170-5060) and the ChemiDoc^TM^ MP Imaging System (BioRad, CA) or West Dure Extended Duration Substrate Kit (AntGene, China) with AI600 Imaging System (GE, USA). Bradford assay measurements were used to normalize the loading of all protein analyses by total protein mass. Specific proteins were detected on the membrane by western blot analysis and quantified using ImageQuant TL 8.1 (GE Healthcare, CA) or Gel-Pro analyzer (LiuYi, China). Each protein was detected using anti-GFP. The level of GFP expression was measured and then normalized using the expression of RecA as an internal control.

## Author contributions

LC designed and coordinated the overall project. SC performed strain construction, RACE, and western blotting. KH performed growth curves and analysis. WS constructed the FLAG-tag labeled mutants for *hfq* and 5′UTR-deleted *hfq* gene, and WS and RL did western blotting and flow cytometry. CB performed flow cytometry. SY constructed pEZ-Tet-GFP plasmid, helped with mutant construction and data interpretation, and revised the manuscript. All authors read and approved the final manuscript.

### Conflict of interest statement

The authors declare that the research was conducted in the absence of any commercial or financial relationships that could be construed as a potential conflict of interest.

## Supplementary material

Figure S1 Structural analysis of UTR candidates using LocARNA.

Figure S2 Experimental analysis of 5′UTRs by RT-PCR.

Figure S3 Summary for the results of 5′ RACE.

Figure S4 Flow cytometry histograms for screen of all UTR-GFP constructs response to stress.

Figure S5 The effect of acetate **(A)** and xylose **(B)** on 5′UTRs.

Figure S6 Growth curves of the wild type and ΔUTR_ZMO0347 strains under a range of ethanol stresses.

Table S1 List of 101 initial candidates and their features.

Table S2 Final 5′UTR candidate lists with features and sequences.

Table S3 List of primers used in this study.
