# Supplementary figures and images for: Identification and Characterization of 5′ Untranslated Regions (5′UTRs) in Zymomonas mobilis as Regulatory Biological Parts
Source: Front Microbiol. 2017 Dec 8;8:2432. doi: 10.3389/fmicb.2017.02432 (PMC5770649; doi:10.3389/fmicb.2017.02432)

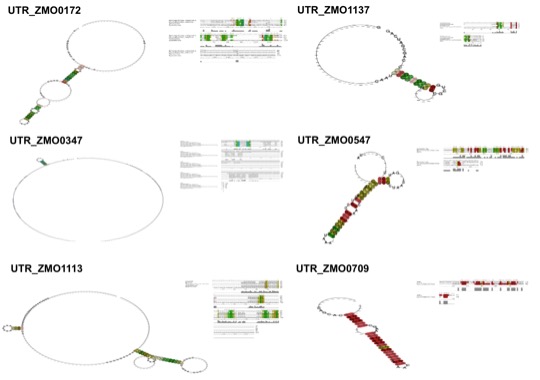

Supplement: Figure S1 — Structural analysis of UTR candidates using LocARNA. [file Image1.JPEG]

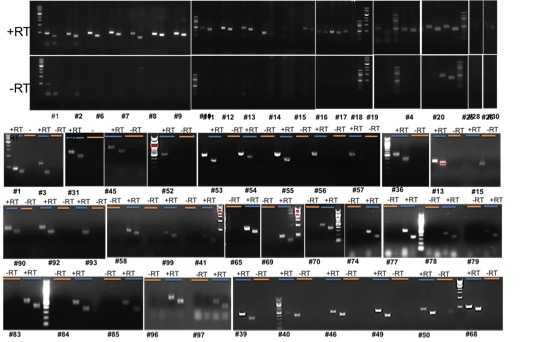

Supplement: Figure S2 — Experimental analysis of 5′UTRs by RT-PCR. [file Image2.JPEG]

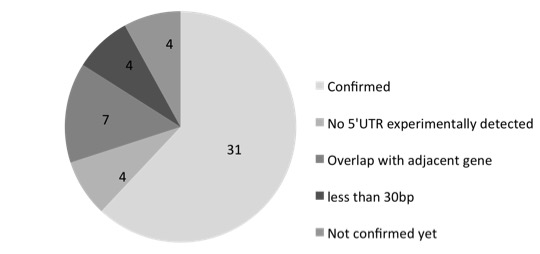

Supplement: Figure S3 — Summary for the results of 5′ RACE. [file Image3.JPEG]

Supplementary Figure 5. The effect of acetate (A) and xylose (B) on 5' UTRs

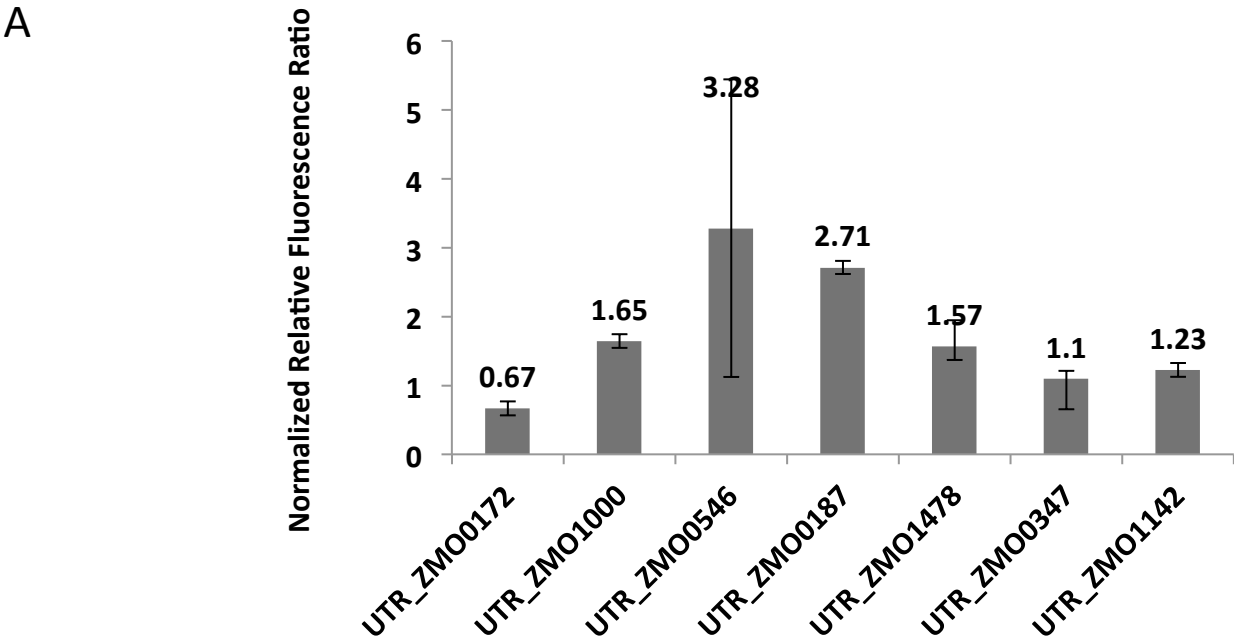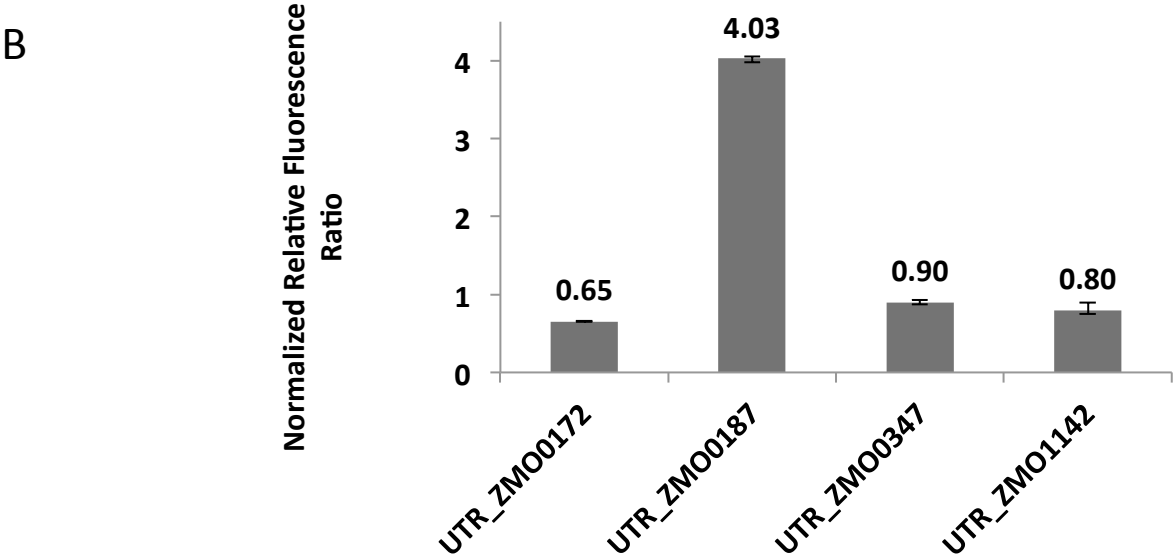

Supplement: Figure S5 — The effect of acetate (A) and xylose (B) on 5′UTRs. [file Image5.PDF]
