# Supplementary material for: Identification and Characterization of 5′ Untranslated Regions (5′UTRs) in Zymomonas mobilis as Regulatory Biological Parts
Source: Front Microbiol. 2017 Dec 8;8:2432. doi: 10.3389/fmicb.2017.02432 (PMC5770649; doi:10.3389/fmicb.2017.02432)

**Supplementary Figure 4. Flow cytometry histograms for screen of all UTR-GFP constructs response to stress.**

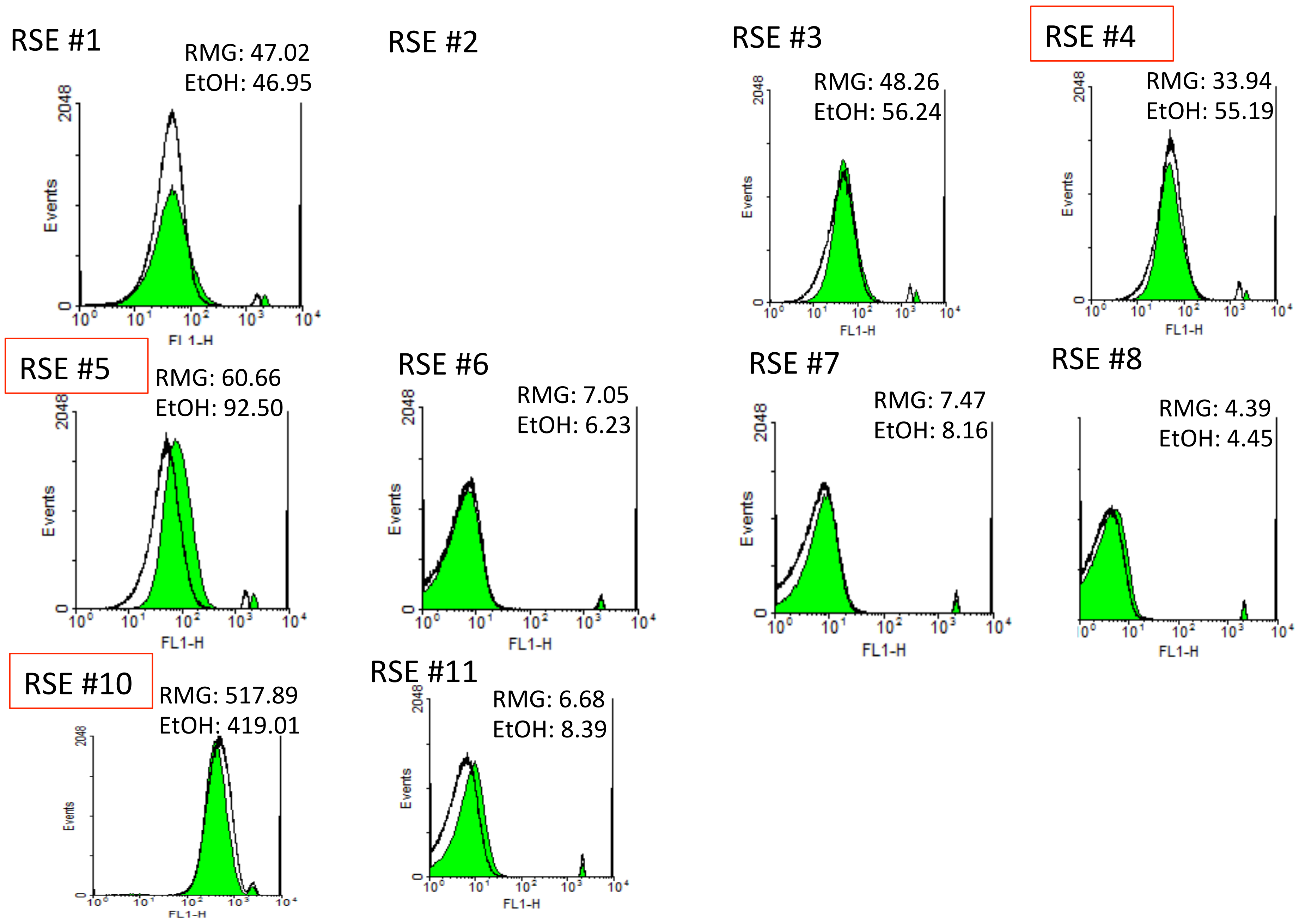

**Supplementary Figure 4. Flow cytometry histograms for screen of all UTR-GFP constructs response to stress.**

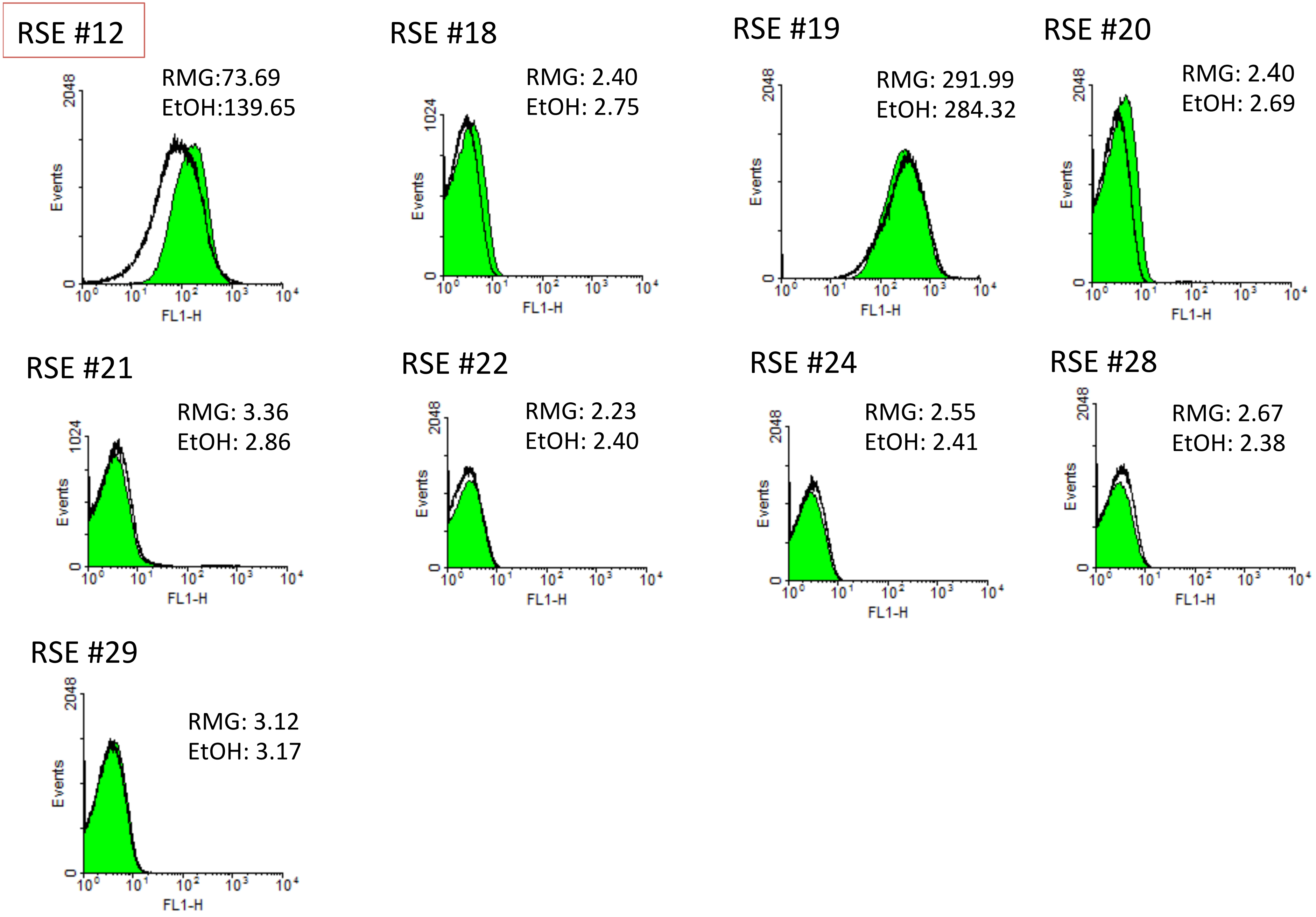

**Supplementary Figure 4. Flow cytometry histograms for screen of all UTR-GFP constructs response to stress.**

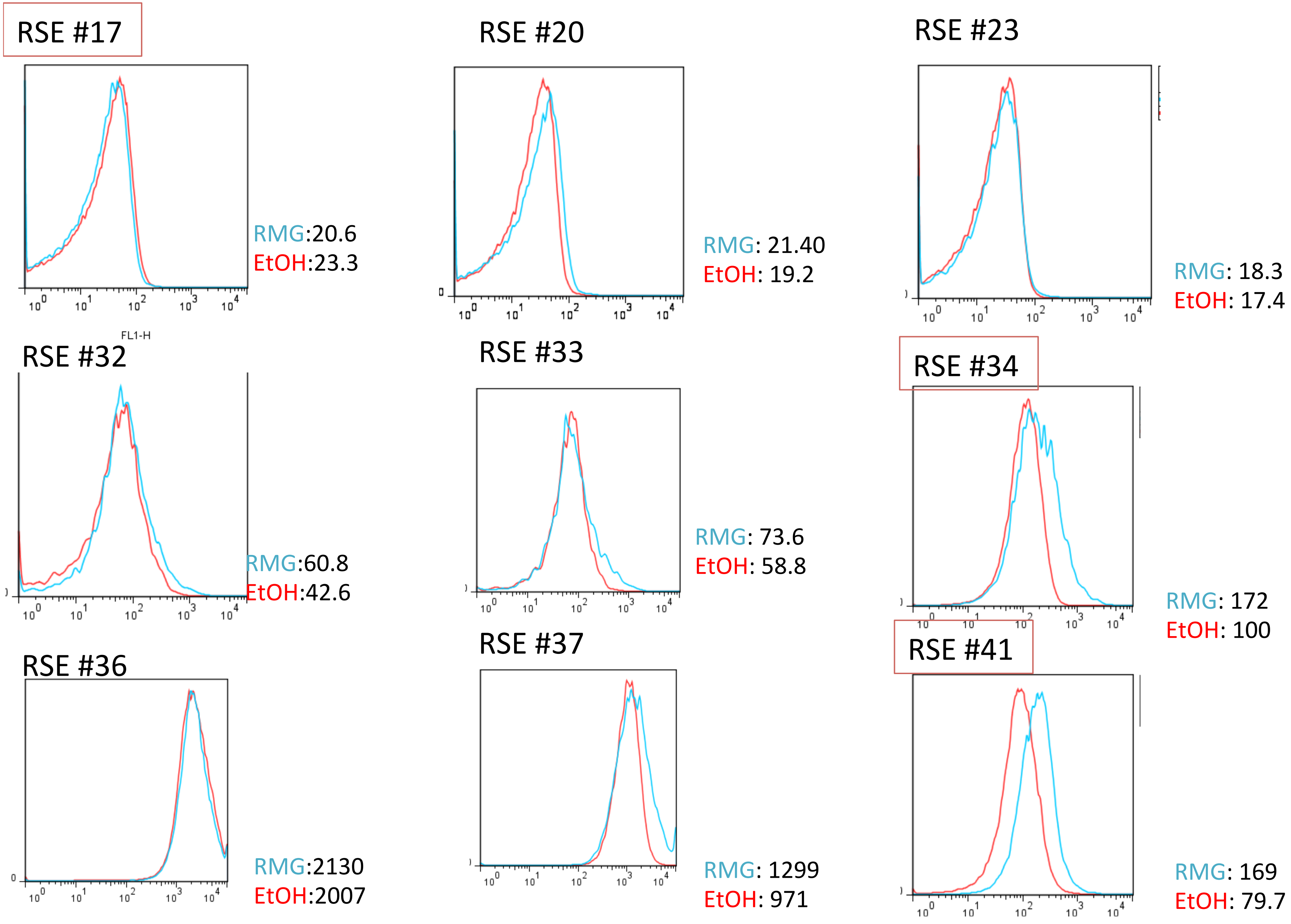

**Supplementary Figure 4. Flow cytometry histograms for screen of all UTR-GFP constructs response to stress.**

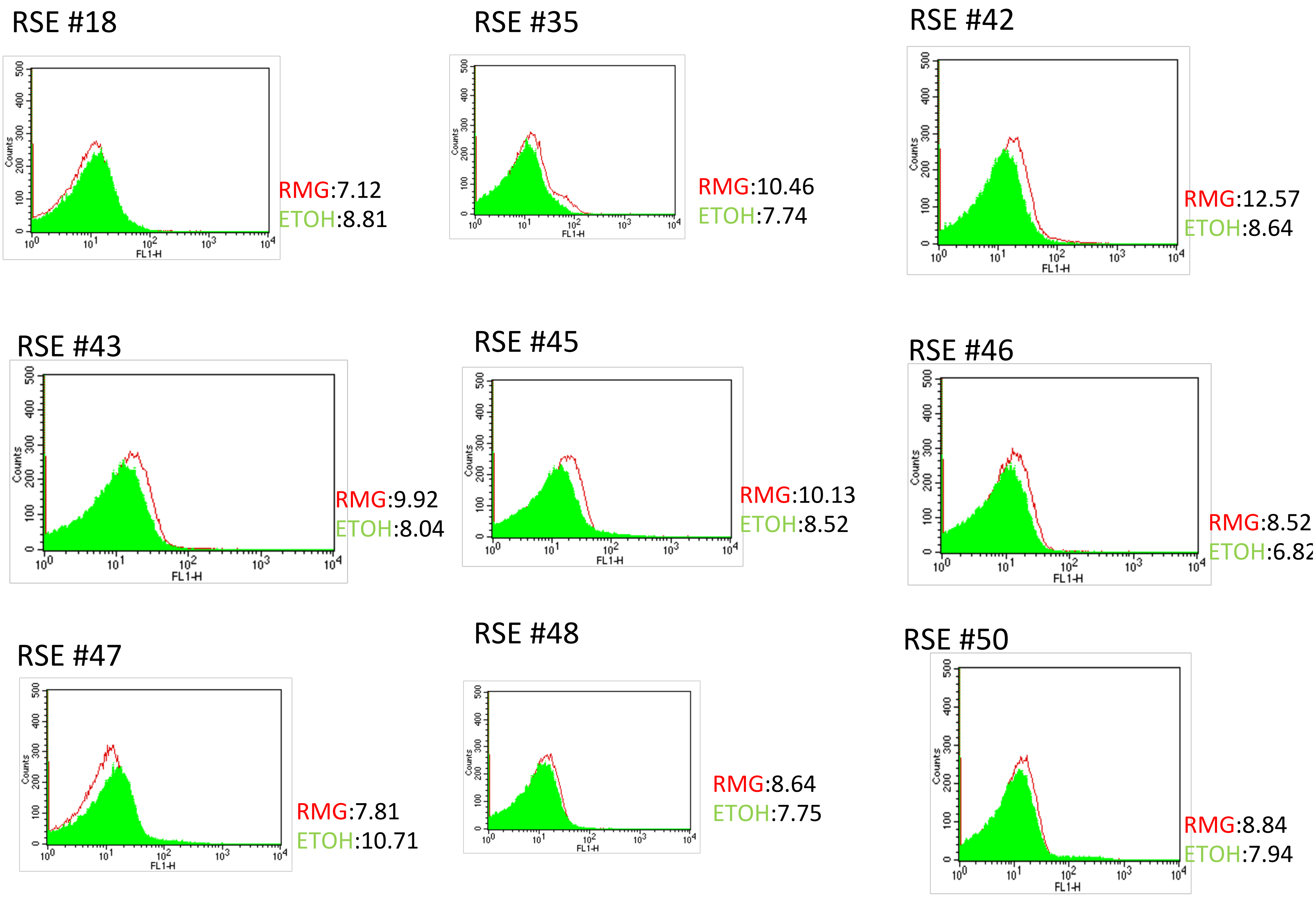

Supplement: Figure S4 — Flow cytometry histograms for screen of all UTR-GFP constructs response to stress. [file Image4.PDF]
