# Supplementary material for: Identification and Characterization of 5′ Untranslated Regions (5′UTRs) in Zymomonas mobilis as Regulatory Biological Parts
Source: Front Microbiol. 2017 Dec 8;8:2432. doi: 10.3389/fmicb.2017.02432 (PMC5770649; doi:10.3389/fmicb.2017.02432)

Supplementary Figure 6. Growth curves of the wild type and ΔUTR_ZMO0347 strains under a range of ethanol stresses.

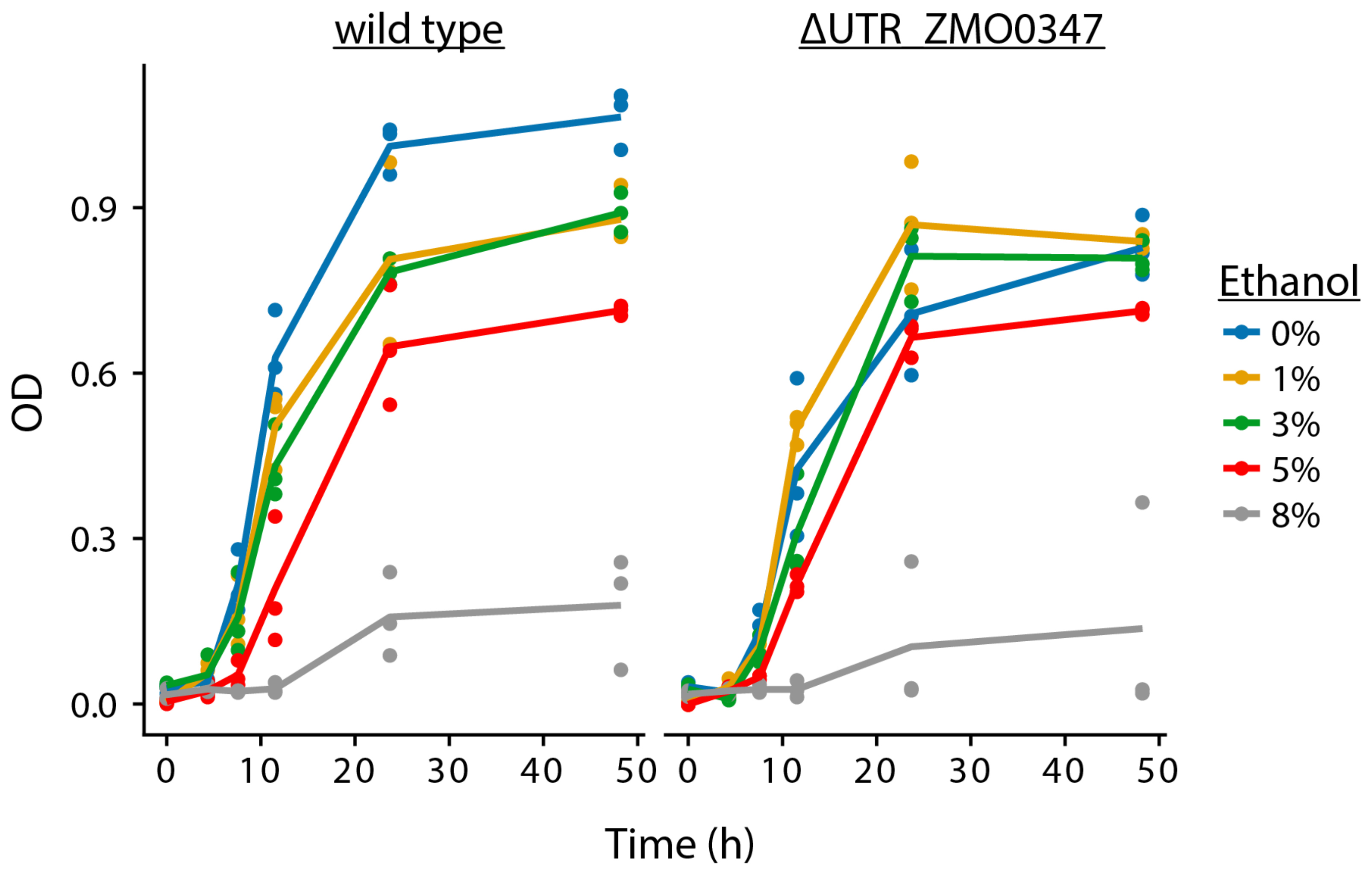

Supplement: Figure S6 — Growth curves of the wild type and ΔUTR_ZMO0347 strains under a range of ethanol stresses. [file Image6.PDF]
